# *Vibrio parahaemolyticus* Infection in Mice Reduces Protective Gut Microbiota, Augmenting Disease Pathways

**DOI:** 10.3389/fmicb.2020.00073

**Published:** 2020-01-30

**Authors:** Rundong Wang, Yijia Deng, Qi Deng, Dongfang Sun, Zhijia Fang, Lijun Sun, Yaling Wang, Ravi Gooneratne

**Affiliations:** ^1^College of Food Science and Technology, Guangdong Ocean University, Guangdong Provincial Key Laboratory of Aquatic Product Processing and Safety, Key Laboratory of Advanced Processing of Aquatic Products of Guangdong Higher Education Institution, Zhanjiang, China; ^2^School of Chemistry and Chemical Engineering, Key Laboratory of Clean Energy Materials Chemistry of Guangdong Higher Education Institutes, Lingnan Normal University, Zhanjiang, China; ^3^Department of Wine, Food and Molecular Biosciences, Faculty of Agriculture and Life Sciences, Lincoln University, Lincoln, New Zealand

**Keywords:** pathogenesis, *Vibrio parahaemolyticus* infection, gut microbiota, 16S rRNA gene, gene sequencing

## Abstract

*Vibrio parahaemolyticus* (Vp), a major food-borne pathogen, is responsible for severe infections such as gastroenteritis and septicemia, which may be accompanied by life-threatening complications. While studies have evaluated factors that affect the virulence of the pathogen, none have investigated the interaction of Vp with gut microbiota. To address this knowledge gap, we compared the effect of Vp on gut bacterial community structure, immunity, liver and kidney function, in pseudo germ-free (PGF) mice and normal (control) mice. Significant damage to the ileum was observed in normal mice compared with the PGF mice. The inflammatory factors IL-1β, IL-6, and TNF-α in normal mice were ∼2.5-fold higher than in the PGF mice, and liver (ALT, AST, ALP) and kidney (BUN) function indices were ∼1.6-fold higher. The Vp infection substantially reduced species composition and richness of the gut microbial communities. In particular, there was a shift in keystone taxa, from protective species of genera *Bacteroides*, *Lactobacillus*, *Bifidobacterium*, and *Akkermansia* in the gut of control mice to opportunistic pathogens *Enterobacteriaceae*, *Proteus*, *Prevotella*, and *Sutterella* in Vp-infected mice, thus affecting microbiota-related biological functions in the mice. Specifically, pathways involved in infectious diseases and ion channels were significantly augmented in infected mice, while the pathways involved in metabolism, digestion and cell growth declined. We propose that the normal mice are more prone to Vp infection because of the alteration in gut-microbe-mediated functions. All these effects reduce intestinal resistance, with marked damage to the gut lining and pathogen leakage into the blood culminating in liver and kidney damage. These findings greatly advance our understanding of the mechanisms underlying interactions between Vp, the gut microbiota and the infected host.

## Introduction

*Vibrio parahaemolyticus* (Vp) is a Gram-negative halophilic bacterium that is widely disseminated in estuarine environments. Vp has been found to be responsible for 20–30% of food poisoning cases in Japan and seafood borne infections in many Asian countries (Letchumanan et al., 2014). Vp also recognized as the leading cause of human foodborne infection from seafood consumption in the United States (Su and Liu, 2007; Shinoda, 2011). The characteristics of Vp infection include abdominal pain, acute gastroenteritis, and septicemia, which in its most severe form (10 to 15% of sufferers) can present with life-threatening complications such as hemolytic uremic syndrome and liver damage (Ryan, 1976; Mertens et al., 1979; Daniels et al., 2000). Most outbreaks of food poisoning are caused by strains of the serotype O3: K6. Vp infections occur mostly via the ingestion of contaminated raw, undercooked, or mishandled marine products (Matsumoto et al., 2000; Newton et al., 2012; Zarei et al., 2012). The main virulence factors that are thermostable direct hemolysin (TDH)-a pore forming protein that contributes to the cytotoxicity and enterotoxicity of Vp in host, and TDH related hemolysin, which plays a similar role as TDH in the disease pathogenesis (Broberg et al., 2011). In addition, Vp also encodes adhesion and type III secretion systems to ensure its survival in the host vivo environment (Dean, 2011; Raghunath, 2015; Li et al., 2019). However, these virulence factors alone cannot directly damage the liver and kidney (Wang et al., 2016). Hence, further research and explanation need to be sought for why the clinical outcomes vary following people’s exposure to Vp.

The gut microbiota performs a diversity of important functions in the host, including promoting the development of the immune system, stimulating metabolic function and repressing pathogenic bacteria (Hooper et al., 2012). There are two major phyla that dominate the human gut- *Bacteroidetes* and *Firmicutes* phyla. The *Bacteroidetes* phyla constitutes of *Bacteroides* and *Prevotella* species, while the *Firmicutes* is made up of *Lactobacillus* and *Clostridium* species (Torres et al., 2001). It has been indicated that there is a correlation between human diseases and bacterial communities in the gut (Turnbaugh et al., 2008). *Vibrio cholerae* can cause explosive diarrhea in which extensive disruption of the intestinal microbes promotes infection by this bacterium (Midani et al., 2018). Microbiota sampled in travelers showed a higher risk of *Campylobacter* infection for those with a specific microbial profile (Kampmann et al., 2016). Diarrheagenic *Escherichia coli* can induce changes in the gut microbiota and distinctive components of these can alter how virulence genes are expressed, thus promoting the infection process (Curtis et al., 2014; Singh et al., 2015). Thus, these compelling evidence suggested that bacterial communities in the host gut may contribute to the pathogenesis of enteric infections. Vp is a significant intestinal pathogen but whether specific gut microbiota in a host resist or promote infection is not known. To advance our understanding of Vp pathogenesis, further research is needed on interactions between gut bacterial communities and Vp.

The term “pseudo germ-free (PGF) mice” refers to mice demonstrably free from intestinal microbes as a result of treatment with mixed antibiotics (Liu et al., 2012) and such mice are an invaluable experimental tool for examining interactions between disease in a host and its gut microbiota (Gordon and Pesti, 1971; Lee et al., 2012; Grover and Kashyap, 2014). Due to recent advances in the application of next generation sequencing technology, it is now possible to gradually unveil the role of the gut microbiome in disease development (Fraher et al., 2012). Use of 16S rRNA gene sequencing to determine the abundance of microbes in different treated samples has also promoted the identification of known and potentially new microorganisms (Yarza et al., 2014).

The main aim of the study was to elucidate the relationship between host gut bacterial communities and Vp infection and advance our understanding of Vp pathogenesis and to develop novel interventions for disease prevention. To achieve this we constructed a PGF mouse as an animal model to evaluate the role of gut microbiota in the Vp pathogenicity *in vivo*. In addition we used 16S rRNA gene sequencing to help identify gut microbiota that could serve as biomarkers of Vp pathogenicity.

## Materials and Methods

### Animals

All experimental protocols and animal handling procedures were carried out in strict accordance with the recommendations in the Guide for the Care and Use of Laboratory Animals (National Institutes of Health Publication No. 80–23, revised 1996). This study was approved by the Ethical Committee on Animal Experimentation of the College of Food Science and Technology, Guangdong Ocean University (No. SYXK2014-0053).

Two-month-old male specific-pathogen-free (SPF) C57BL/6J mice (20 ± 5 g) were obtained from the animal center of Guangdong Province. The mice were housed in stainless steel cages and allowed to acclimatize to environmental conditions for 5 days before experiments. The animals had free access to distilled water and sterilized food; the room temperature was maintained at 20 ± 2°C with a relative humidity of 60 ± 5% on a 12 h light–dark cycle. The mice were randomly allocated to four groups for treatment: control SPF mice, PGF mice, SPF mice infected with Vp, and PGF mice infected with Vp.

The procedure for preparing PGF mice was based on previous studies (Clarke et al., 2010; Wang et al., 2011; Ge et al., 2017) but modified to deliver antibiotics by gavage rather than in the animals’ drinking water. This was done to ensure consistency of exposure. The SPF C57BL/6J mice in the PGF group received a mixture of non-absorbable antibiotics – vancomycin (200 mg/kg), metronidazole (200 mg/kg), and neomycin (300 mg/kg) by orally gavage twice a day for 7 days. Each mouse was housed in a micro-isolator cage under germ-free conditions with free access to sterilized water and food.

### Evaluation of Antibiotic Treatment on Serum and Gut Flora

Tests to evaluate the pseudo-germ-free model in comparison to normal SPF C57BL/6J mice (controls) were carried out on days 3 and 6 after the final oral gavage with of mixed antibiotics. The same procedure was employed for control mice as is described below for the model mice.

Five pseudo-germ-free mice were euthanized by exsanguination following ether vapor narcosis (via a funnel) after overnight fasting. Blood was sampled by percutaneous cardiac puncture. Half the volume of blood collected was centrifuged at 3,500 rpm for 10 min and the serum used to assess physiological function. Detection kits (Nanjing Jiancheng Bioengineering Institute, Nanjing, China) were used to measure three liver-specific enzymes [aspartate aminotransferase (AST), alanine aminotransferase (ALT), and alkaline phosphatase (ALP)] and the kidney-specific index, blood urea nitrogen (BUN). The remaining blood was transferred into tubes containing EDTA for measurement of red blood cells (RBC), white blood cells (WBC), hemoglobin (Hb), and platelet concentrations, using an automatic hematology analyzer (Cell – DYN 3700, Abbott Diagnostics, Abbott Park, IL, United States).

The ileum and colon contents were collected following the blood collection. The mice were sacrificed by cervical dislocation and dissected under a biosafety hood. The ileum was examined for damage, flushed with ice-cold sterilized PBS, and the ileal contents collected aseptically. Samples were placed in 1.5-mL sterile tubes, snap-frozen on dry ice, and stored at −80°C. The numbers of microbiota were measured by a plate counting method as described below.

The samples were weighed, mixed with sterile 0.01 M phosphate-buffered saline solution (PBS, pH 7.2) at 1 g: 9 mL ratio and homogenized for 30 s in a Stomacher^®^ in a sterile environment. The homogenates were diluted (10×) in sterile PBS solution, and a 100-μL volume of homogenate was plated onto selective media in triplicate and incubated at 37°C for 24 h (for aerobes) or 48 h (for anaerobes). Colony forming units (CFU) were counted manually to determine the density of viable cells in each sample (expressed as CFU/g). The triplicates were analyzed independently with the whole process completed within 20 min.

The choice of selective media was based on the composition of dominant microbiota in ileum and colon. In this test, we used *Bifidobacterium*, *Lactobacillus*, *E. coli*, and *Enterococcus* to characterize the gut flora (Hooper and Gordon, 2001; Qin et al., 2010). For aerobes, *E. coli* and *Enterococcus* were detected using eosin methylene blue and enterococcus chromogenic media. For anaerobes, *Bifidobacterium* and *Lactobacillus* were detected by TPY agar and MRS agar media and incubated in an anaerobic tank.

### Culture of *V. parahaemolyticus* and Infection of Mice

*Vibrio parahaemolyticus* ATCC 33847 is a typical pathogen, containing *tdh* and *tlh* genes, two kinds of type of III secretion systems (T3SS1 and T3SS2) and the T6SS2 that co-exists with T3SS2 system, was used in this study. The Vp was stored in 25% glycerol at −20°C before being grown at 37°C for 24 h in Luria Broth (LB; BLBT, Beijing, China) containing 3% NaCl. The inoculum was thrice passaged in LB-3% NaCl. The final density of inoculant was adjusted to ∼10^9^ CFU/mL and used to infect mice.

Mice were fasted overnight then randomly divided into three groups of 5 mice each: control SPF mice, PGF model mice and SPF mice for inoculation. The PGF and SPF mice were gavaged with a suspension of Vp (as described above) at 0.2 mL/10 g body weight (bw). The control group was gavaged with fresh sterilized LB medium. The three groups of mice were observed for 12 h before sample collection. This *in vivo* experiment was performed in triplicate.

### Blood and Ileum Content Samples

The mice were anesthetized with ether, as described in section “Evaluation of Antibiotic Treatment on Serum and Gut Flora,” and blood samples collected by cardiac puncture. The mice were sacrificed by cervical dislocation and dissected under a biosafety hood. The ileum was examined for any damage, flushed with ice-cold sterilized PBS, and the ileal contents collected aseptically and stored at −80°C for gut microbiota analyses.

The blood samples were centrifuged at 3,500 rpm for 10 min and serum collected. Three liver-specific enzymes (AST, ALT, and ALP) and the kidney-specific index BUN were detected using kits (Nanjing Jiancheng Bioengineering Institute, China). The pro-inflammatory markers [Interleukin-1β (IL-1β), Interleukin-6 (IL-6), and Tumor Necrosis Factor (TNF-α)] and an anti-inflammatory marker [(Interleukin-10 (IL-10)] were determined by enzyme-linked immunosorbent assay (ELISA) kits (Neobioscience Technology Co., Shenzhen, China) according to the manufacturer’s protocols.

To better understand the effect of Vp infection on the richness and species composition of the gut microbiota, high-throughput sequencing of the V3–V4 region of the 16S rRNA gene was used. The metagenomic DNA was extracted from ileal samples using the Power Max DNA isolation kit provided by MoBio Laboratories, Carlsbad, CA, following to the manufacturer’s recommendations, and quantified by UV spectroscopy (NanoDrop ND-1000 spectrophotometer, Thermo Fisher Scientific, Waltham, MA, United States). The V3–V4 region of the 16S rRNA gene was amplified from metagenomic DNA using a pair of universal primers (515F 5′-GTGCCAGCM GCCGC GGTAA-3′, 806R 5′-GGACTACHVGGGTWTCTAAT-3′) (Shang Q. et al., 2017). The PCR products of each sample were checked and purified using Agencourt AMPure XP Beads (Beckman Coulter, Indianapolis, IN, United States) and the PicoGreen dsDNA Assay Kit (Invitrogen, Carlsbad, CA, United States). Equimolar amounts of PCR products were pooled and sequenced using the Illumina HiSeq4000 platform at GUHE Info Technology Co. (Hangzhou, China).

The QIIME software package (version 1.9.0) was employed to filter raw sequencing reads in terms of quality using the script, as previously described (Caporaso et al., 2010; Shang Q. et al., 2017).

Vsearch (v1.11.1) was applied to pick the operational taxonomic units (OTUs) at an identity of 97% (Rognes et al., 2016). A Venn diagram was constructed to compare the compositional OTUs of the bacterial communities in each group by using R packages (v3.2.0) as previously documented (Zaura et al., 2009).

Operational taxonomic unit-level alpha diversity indices, such as Chao1, Ace, Simpson index and Shannon index for evaluation of community richness and species composition, were calculated by Mothur software packages (v1.30.1) (Ling et al., 2014).

Principal coordinate analysis (Ramette, 2007) was performed to compare the structural differences in microbial communities. Principal components 1 (PC1) and 2 (PC2) were subjected to the Wilcoxon rank-sum test to determine the significance of differentiation of microbiota structure among control mice and Vp-infected mice.

Linear discriminant analysis Effect Size (LEfSe) was performed to identify and characterize gut microbiota differences between the control group and Vp-infected group, using the online version of Galaxy^3^ (Segata et al., 2011). Linear discriminant analysis (LDA) was performed using a one-against-all strategy; a significance value of less than 0.05 combined with an effect size threshold of 2 was selected.

The OTU table was normalized by the 16S rRNA copy number per chromosome, and the Kyoto Encyclopedia of Genes and Genomes (KEGG) classified functions by Phylogenetic Investigation of Communities by Reconstruction of Unobserved States (Langille et al., 2013; Zhang et al., 2019) using the Galaxy platform^1^. This process was conducted to obtain the metabolic pathways of bacteria. The significant differences in KEGG pathways between Vp-infected mice and control mice were tested using the Statistical Analysis of Metagenomic Profiles software package (v2.1.3) (Parks et al., 2014), and results shown by heatmap.

### Statistical Analysis

All data were analyzed using the software SPSS 19.0 (SPSS Inc., Chicago, IL, United States). Differences between the means and changes in the diversity of gut bacterial community were compared by one-way analysis of variance, followed by Tukey’s *post hoc* test, with the level of significance set at *p* < 0.05.

## Results

### Evaluation of Antibiotic Treatment

The effect of mixed antibiotics on the gut microbiota of mice was evaluated by a plate counting method with media selective for aerobes and anaerobes. The dominant intestinal microbiota in the PGF mice were significantly reduced in density by the mixed antibiotic treatment compared with untreated mice (Table 1). On day 3 following the last oral gavage, there was no *Bifidobacterium* and *Lactobacillus*, and the numbers of *E. coli* (1.01 ± 0.02 log_10_ CFU/g) and *Enterococcus* (0.12 ± 0.01 log_10_ CFU/g) were very significantly lower (*p* < 0.01) than in the control group. In the PGF mice, the gut microbiota was almost completely eliminated and there was no sign of recovery on day 6 when sampling results were similar to day 3.

**TABLE 1:** Dominant gut microbiota in normal mice and pseudo germ-free mice (log_10_ CFU/g, *n* = 5)^+^.

**Time point of detection after final mixed antibiotic treatment**	**Dominant microbiota**	**Normal**	**Pseudo germ-free**
Day 3	*Bifidobacterium*	8.83 ± 0.25	0.00 ± 0.00**
	*Lactobacillus*	7.97 ± 0.34	0.00 ± 0.00**
	*Escherichia coli*	7.68 ± 0.39	1.01 ± 0.02**
	*Enterococcus*	8.17 ± 0.28	0.12 ± 0.01**
Day 6	*Bifidobacterium*	8.62 ± 0.33	0.00 ± 0.00**
	*Lactobacillus*	8.02 ± 0.17	0.00 ± 0.00**
	*Escherichia coli*	7.81 ± 0.42	1.32 ± 0.06**
	*Enterococcus*	8.24 ± 0.13	0.23 ± 0.03**

^+^Data are expressed as the mean ± standard deviations of three replicates. Means of pseudo germ-free group with **are significantly different from the control group (*p* < 0.01). Normal mice refer to SPF C57BL/6J mice that did not receive the mixed antibiotic treatment.

The effect of mixed antibiotics on physiological functions of mice was assessed by hemogram and serum biochemical indices. There were no significant differences in hematological parameters between normal untreated mice and PGF mice on days 3 or 6 following treatment with mixed antibiotics (Table 2). The concentration of WBC, RBC, Hb, and platelets of PGF mice were not significantly different (*p* > 0.05) to normal mice and neither were the serum biochemical parameters AST, ALT, ALP, and BUN. Therefore, it appears that exposure to mixed antibiotics only removed intestinal microbial flora and did not affect the monitored hematological parameters in mice over 6 days.

**TABLE 2:** Hematological parameters of mice in normal group and pseudo germ-free group (*n* = 5)^+^.

**Time of sampling following the final mixed antibiotic treatment**	**Hematological indices**		**Normal**	**Pseudo germ-free**
Day 3	Blood indices	WBC (×10^9^⋅liter^–1^)	6.77 ± 1.23	7.81 ± 1.37
		RBC (×10^12^⋅liter^–1^)	10.26 ± 2.07	11.31 ± 1.76
		Hb (g⋅liter^–1^)	145.61 ± 17.88	140.26 ± 16.92
		Platelets (×10^11^⋅liter^–1^)	8.36 ± 0.94	9.13 ± 1.41
	Serum biochemical indices	ALP (U⋅liter^–1^)	71.28 ± 11.04	76.29 ± 10.16
		AST (U⋅liter^–1^)	119.01 ± 18.57	114.88 ± 15.32
		ALT (U⋅liter^–1^)	56.24 ± 5.81	58.49 ± 6.17
		BUN (mmol⋅liter^–1^)	9.97 ± 3.69	9.12 ± 1.73
Day 6	Blood indices	WBC (×10^9^⋅liter^–1^)	7.13 ± 1.69	8.01 ± 2.03
		RBC (×10^12^⋅liter^–1^)	11.12 ± 4.53	12.16 ± 3.88
		Hb (g⋅liter^–1^)	149.11 ± 12.35	145.36 ± 11.51
		Platelets (×10^11^⋅liter^–1^)	10.61 ± 1.23	11.26 ± 1.14
	Serum biochemical indices	ALP (U⋅liter^–1^)	70.16 ± 10.13	74.32 ± 9.37
		AST (U⋅liter^–1^)	108.11 ± 12.15	110.91 ± 10.42
		ALT (U⋅liter^–1^)	54.39 ± 6.11	57.42 ± 7.32
		BUN (mmol⋅liter^–1^)	10.26 ± 1.03	9.99 ± 0.89

*^+^Data are shown as the mean ± standard deviation of three replicates. Normal mice refer to SPF C57BL/6J mice that did not receive the mixed antibiotic treatment.*

### Effects of Vp Infection on Ileum, Liver and Kidney Function and Inflammation

**FIGURE 1:**
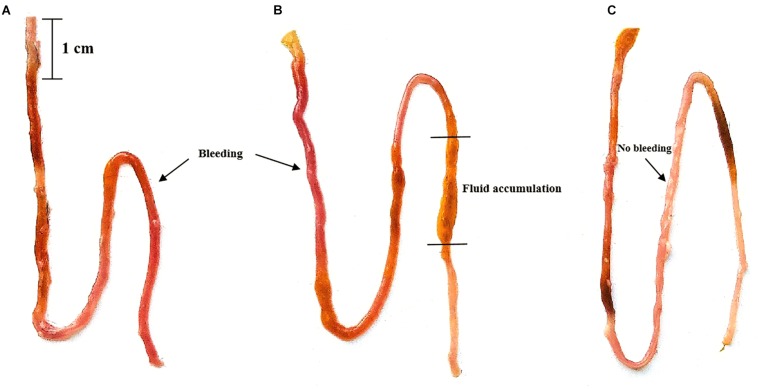
Effects of oral gavage with *Vibrio parahaemolyticus* (Vp) on the ileum of pseudo germ-free and normal (SPF) mice. **(A)** Pseudo germ-free mouse, **(B)** SPF mouse, **(C)** control mouse. The control mice were given fresh sterilized LB medium instead of Vp.

The ileal damage differences were compared by observing ileal fluid accumulation and ileal wall bleeding. When the two groups of mice were administered Vp, both the PGF group and normal (SPF) mice showed ileal bleeding, ileal fluid and intestinal damage compared with the untreated controls and these effects were most marked in normal SPF mice (with intact gut microbiota). The ileal damage caused by Vp in PGF mice and normal mice is shown in Figure 1.

**FIGURE 2:**
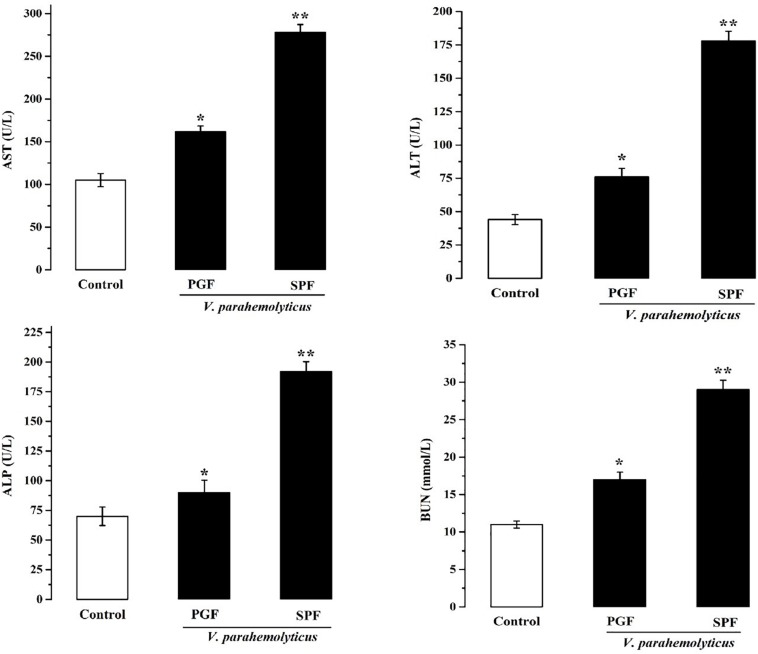
Effects of *Vibrio parahaemolyticus* on serum AST, ALT, ALP, and BUN indices of pseudo germ-free (PGF) and normal (SPF) mice. Data are expressed as the mean ± SEM (*n* = 5) compared with the control group gavaged with fresh sterilized LB medium. *p < 0.05; **p < 0.01.

Damage to the metabolism of mice caused by Vp may be revealed by liver and kidney function tests. Therefore, serum biochemical parameters indicative of liver and kidney function were measured at 12 h in normal SPF and PGF mice gavaged with Vp (Figure 2). AST, ALT, ALP, and BUN were significantly elevated (*p* < 0.05) in infected SPF and PGF groups compared with the control group. Moreover, AST, ALT, ALP, and BUN activities in the SPF group were 1.5, 1.7, 1.9, and 1.4 times higher, respectively, than in the PGF group.

**FIGURE 3:**
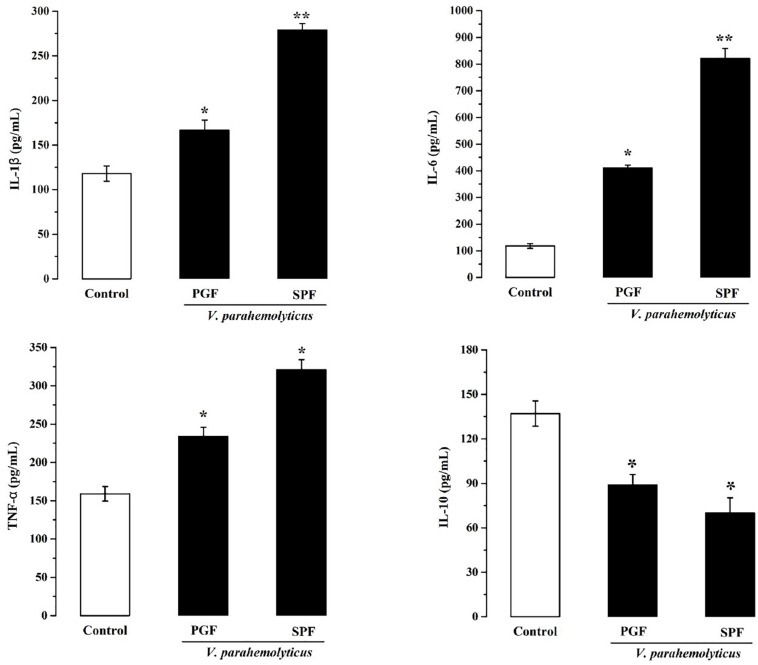
Effects of *Vibrio parahaemolyticus* on the serum IL-1β, IL-6, TNF-α, and IL-10 indices of pseudo germ-free mice and normal mice. Data are expressed as the mean ± SEM (*n* = 5) compared with the control group gavaged with fresh sterilized LB medium. *p < 0.05; **p < 0.01.

Inflammation is the body’s response to infection. The pro-inflammatory and anti-inflammatory marker studies showed that IL-1β, IL-6, and TNF-α were significantly increased (*p* < 0.05) and that IL-10 was significantly reduced (*p* < 0.05) in the Vp-infected SPF and PGF groups compared with the control group (Figure 3). In the SPF group, the pro-inflammatory markers IL-1β, IL-6, and TNF-α were 1.6, 4.4, and 1.4 times that of the PGF group, and the anti-inflammatory marker IL-10 was approximately half that in the PGF mice.

### Effects of Vp Infection on Gut Microbial

To better understand the effect of Vp infection on the richness and species composition of the gut microbiota, high-throughput sequencing of the V3–V4 region of the 16S rRNA gene was used. The pyrosequencing coverage of more than 99.1% indicates an adequate sequencing depth for all samples (Table 3). To estimate bacterial richness and composition of the gut microbiota, the Chao1, Ace, Shannon and Simpson indices were calculated (Table 3). Both richness and composition of the gut microbiota were reduced following Vp infection and these differences were statistically significant (*p* < 0.05). These preliminary results suggest that Vp infection could alter the community structure of gut microbiota.

**TABLE 3:** Evaluation of community richness and species composition (Chao1, Ace, Shannon and Simpson indices) of mouse gut microbiota after *V. parahaemolyticus* (Vp) infection^+^.

**Groups**	**Shannon**	**Chao1**	**Ace**	**Simpson**	**Coverage**
Control	6.01 ± 0.22	767 ± 28.01	758 ± 26.18	0.044 ± 0.01	0.99 ± 0.01
Vp-infected	4.44 ± 0.25*	691 ± 22.48*	681 ± 20.01*	0.085 ± 0.03*	0.99 ± 0.01

^+^Values are mean ± SEM Means of the Vp-infected group (SPF C57BL/6J mice gavaged with Vp suspension) with *are significantly different from the control group (*p* < 0.05) gavaged with fresh LB medium.

**FIGURE 4:**
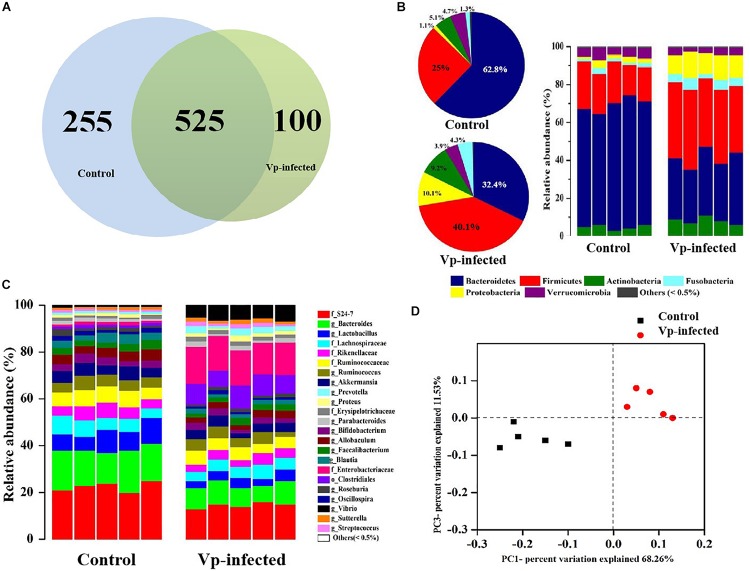
*Vibrio parahaemolyticus*-induced gut microbiota dysbiosis. **(A)** Venn diagram analysis of unique/shared OTUs in the gut microbiota of experimental mice following *V. parahaemolyticus* (Vp) infection. **(B,C)** The ileum microbiome composition profiles at the phylum and genus level in the control group and Vp-infected groups (each color represents one bacterial taxon). **(D)** UniFrac principal co-ordinates analysis estimates of ileum microbiota in the control and Vp-infected groups.

To analyze the microbiota in ileal contents, sequences with at least 97% similarity were combined into OTUs. As shown in a Venn diagram, 780 OTUs were identified in ileal contents of the control group. In the Vp-infected groups 255 of these OTUs decreased and 100 were different from the controls (Figure 4A). Overall, 32.7% of ileal OTUs changed following Vp infection. At the phylum level (Figure 4B), *Bacteroidetes*, *Firmicutes*, *Fusobacteria*, *Actinobacteria*, *Proteobacteria* (which includes Vp), and *Verrucomicrobia* were most abundant in the ileum, making up 62.8, 25.0, 1.3, 5.1, 1.1, and 4.7% of all identified OTUs, respectively. The proportions of *Bacteroidetes* and *Verrucomicrobia* were decreased in the ileum following Vp infection whereas *Firmicutes*, *Fusobacteria*, *Actinobacteria*, and *Proteobacteria* were increased. At the genus level (or family level where microbe could not be identified to genus) (Figure 4C), the abundance of *Enterobacteriaceae*, *Clostridiales*, *Prevotella*, and *Vibrio* increased while *S24_7*, *Bacteroides*, *Lactobacillus*, *Bifidobacterium*, and *Akkermansia* decreased following Vp infection. In addition, at the genus level, abundance of *Rikenellaceae*, *Ruminococcus*, and *Faecalibacterium* were also significantly different from the control group (data not shown). Moreover, according to the UniFrac principal coordinates analysis, the ileal content samples of the control group clustered in the lower left quadrant, while those of the Vp-infected groups clustered in the upper right quadrant, indicating that gut microbiota changed significantly following exposure to Vp (Figure 4D).

**FIGURE 5:**
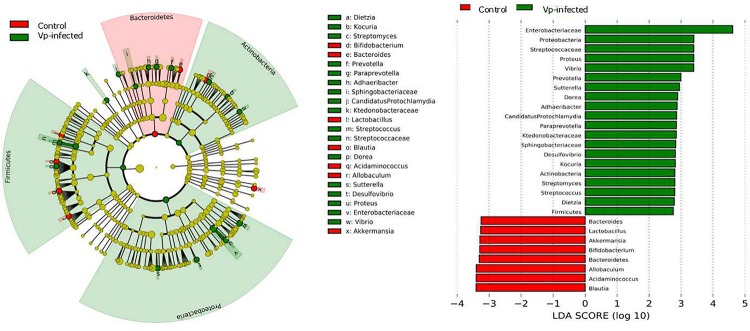
Identification of significant differences in bacterial taxa between Vp-infected and control groups. Cladogram shows the differences and phylogenic location. In each section, the diameter of the circle is proportional to the abundance of the taxon. Ileum microbial communities from Vp-infected and control mice were compared using LEfSe (red = taxon significantly enriched in control; green = taxon significantly enriched in Vp-infected; yellow = not-significant). Histogram of the LDA scores computed for features differentially abundant between the Vp-infected and control groups. LEfSe scores represent the degree of consistent difference in relative abundance between features in two groups of analyzed microbial communities. The clades of the histogram identify statistical and biological differences between the communities. Taxonomic groups showing LDA scores >2.0 with *p* < 0.05.

To fully ascertain the effects of Vp on gut microbiota and identify biomarker microbiota that differ between the Vp-infected groups and the control group, we further analyzed the gut microbiota at different taxonomic levels using the LEfSe algorithm with significance set at *p* < 0.05. As shown in the taxonomic cladograms, LEfSe analysis visualized and confirmed the modulatory effects of Vp on the gut microbiota at different taxonomic levels in C57BL/6J mice (Figure 5). Specifically, Vp decreased the *Bacteroidetes*. At genus level, *Bacteroides*, *Lactobacillus*, *Bifidobacterium*, *Blautia*, *Acidaminococcus*, *Allobacullum*, and *Akkermansia* decreased. Among the intestinal microbes that were enriched by Vp – *Enterobacteriaceae*, *Streptococcaceae*, *Proteus*, *Vibrio*, *Prevotella*, *Sutterella*, *Dietzia*, *Adhaeribacter*, *Desulfovibrio*, *Paraprevotella*, *Sphingobacteriaceae*, and *Kocuria* – most belonged to the phyla *Firmicutes*, *Actinobacteria*, and *Proteobacteria*. Thus, the presence of these sensitive taxa may serve to indicate existence of Vp infection.

**FIGURE 6:**
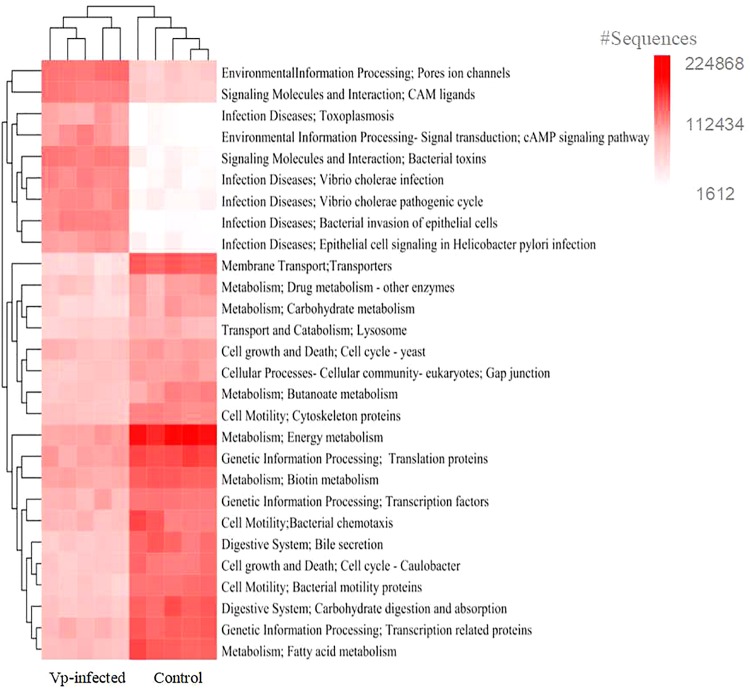
Predicted KEGG pathways of mice gut microbiota. Changes in the abundance of KEGG metabolic pathways in the Vp-infected group relative to the control group at 12 h. The red color intensity indicates the richness of metabolic pathways.

To evaluate whether alterations in gut-bacterial-mediated functions were caused by infection, we compared predicted bacterial KEGG pathways between the control and Vp-infected mice. The results showed that KEGG pathways were significantly influenced by Vp infection (Figure 6). In particular, KEGG pathways known to be involved in pathogen infection, such as infectious diseases (e.g., *V. cholerae* infection, *V. cholerae* pathogenic cycle, bacterial invasion of epithelial cells and epithelial cell signaling in *Helicobacter pylori* infection), ion channel pores, bacterial toxins and the cAMP signaling pathway were significantly induced following Vp infection. In contrast, metabolic functional processes (e.g., drug metabolism-other enzymes, carbohydrate/energy/biotin/butanoate/fatty acid metabolism), digestive activities (e.g., bile secretion, carbohydrate digestion and absorption), cell motility, cell growth and death and genetic information processing, exhibited an opposite pattern.

## Discussion

The Vp pathogen has become more widespread in recent years with more than 50 million people in estuarine areas at risk of Vp infection from unsafe food (Xu et al., 1994; Shinoda, 2011; Romilio et al., 2017; Lee et al., 2018). Although the relationship between virulence factors and pathogenicity is recognized (Vongxay et al., 2008; Hiyoshi et al., 2010; Broberg et al., 2011), we are far from delineating the pathogenesis of Vp in the host intestine. More recent research has reported that the gut microbiota is altered in response to enteric pathogen infections and diseases (Lofgren et al., 2011; Zhang et al., 2012; Tuomisto et al., 2014). Hence, an improved understanding of how the host gut microbiota affects Vp pathogenicity could support the design of better infection prevention methods.

In this study, differences in damage caused by Vp in PGF mice and normal mice were investigated to better understand the role of gut microbiota during Vp infection. In normal mice, inflammation (Figure 3) and damage to the intestines (Figure 1), liver and kidney (Figure 2) were more marked than in PGF mice. Other studies have shown that exogenous enteric pathogens can induce dysbiosis of the gut microbiota resulting in reduced intestinal resistance to harmful bacteria, and increased intestinal inflammation and permeability, providing an opportunity for a systemic infection by these pathogens (Wang et al., 2011; Kamada et al., 2012). *Clostridium difficile* infected patients usually show decreased diversity of gut microbiota and increased facultative anaerobes in the intestines, which together may promote peptic ulcers, gastroenteritis and colitis (Zalig and Rupnik, 2014). It is suggested (Anonymous, 2012) that *Salmonella* could interact with gut microbiota, resulting in dysbiosis and intestinal inflammation, thus providing a growth advantage for *Salmonella*. The resulting damage to the intestinal barrier, could, allow bacterial toxins and pathogens to enter the body, leading to a systemic infection. Thus, we hypothesize that the normal steady-state network interactions between intestinal flora is destabilized by the increased number of Vp, resulting in marked changes to gut microbiota diversity and abundance. This together with the pathogenic Vp itself leads to the induction of markers of both inflammation and liver and kidney damage in our infected mice.

Although large numbers of microbes reside normally in the host intestinal tract, many species of which are beneficial to the host body, there is potential for some undesirable microbiota to affect the intestinal immune system (Furusawa et al., 2013) and cause inflammatory reactions in the host (Garrett et al., 2010). It was apparent that the diversity (richness and composition) of gut microbiota in the ileum declined during Vp infection (Table 2), indicating that Vp can displace some lineages such as *Bacteroidetes* and *Verrucomicrobia* (Figure 4B). According to the diversity resistance hypothesis, a less diverse community has a reduced chance of harboring species with antagonistic traits toward pathogens and vice versa (Kennedy et al., 2002). Also, where gut bacterial diversity is reduced, the vacant niches may be taken up by invading pathogens (Dillon et al., 2005; Mallon et al., 2015; Nie et al., 2017). Similarly, Chen et al. (2011) reported that a reduced abundance of *Bacteroidetes* in parallel with an increase in *Proteobacteria* and *Fusobacteria* species in samples of gut microbiota taken from a diseased host were attended with a reduced gut bacterial diversity compared with healthy controls. 16S rRNA gene sequencing revealed a change in 32.7% of ileal OTUs following Vp infection (Figure 4A), which was significantly different from the control group (Figure 4D). Thus, Vp infections can substantially alter the richness and composition of the bacterial community in the mouse gut, which may create niches for colonization by pathogens because of reduced competition, which, in turn, could possibly induce more serious inflammation, gut epithelial damage, impediment to the growth of the good resident species and proliferation of indigenous opportunistic pathogenic bacteria (Thiennimitr et al., 2011; Ayres et al., 2012; Kamada et al., 2013). Besides, dysbiosis of microbiota composition has been shown to play a contributory role in the pathogenesis and progression of liver and kidney diseases (Chen et al., 2011). This assertion is supported by the present finding that following Vp gavage, normal mice showed higher levels of markers for liver and kidney damage than the PGF mice, possibly due to altered metabolic pathways in the normal gut microbiota resulting in variation in the composition and richness of gut microbiota species.

Given that microbiota in the host gut are sensitive to Vp infection, we identified some microbiota that could potentially be used as biomarkers indicative of infection. Notably, seven normally present OTUs were absent in the Vp-infected group (Figure 5). Among them, affiliated with *Lactobacillus*, *Bifidobacterium*, and *Akkermansia*, are probiotics, which have been shown to protect the host from mucosal inflammation by stimulating the anti-inflammatory cytokine IL-10 or down-regulating inflammatory cytokines (Shang Q.S. et al., 2017). The relative abundance of *Lactobacillus* was significantly decreased compared with *Bifidobacterium* and *Akkermansia* following Vp infection (Figure 4C). *Lactobacillus* can generate reactive oxygen species and produce bacteriocin molecules capable of killing pathogenic microbes (Jones et al., 2012). Other studies have shown this potential to restore gut function by stimulating mucosal and humoral immune responses to eliminate enteropathogens (Wardwell et al., 2011; Berrilli et al., 2012). *Lactobacillus* species also play an important protective role in *Salmonella enterica* infection (Burkholder and Bhunia, 2009). We therefore anticipate that *Lactobacillus* might be used as the biomarker indicating degree of protection against Vp infection. Other protective species may be *Bacteroides*, *Blautia*, and *Allobacullum*, which are involved in the production of short-chain fatty acids, antibacterial peptides and bile acids, all of which can support intestinal resistance to pathogenic bacteria (Kang et al., 2013; Zhang et al., 2016). In contrast, 17 OTUs were increased in response to Vp infection. Among them, *Enterobacteriaceae* including pathogenic species such as *E. coli*, *Shigella* and *Salmonella* (Lupp et al., 2007), *Proteus*, *Prevotella* and *Sutterella* have been linked to inflammation and disease (He et al., 2013; Zackular et al., 2013; Zhang et al., 2015), and in particular *Prevotella* correlates with the severity of liver and kidney diseases. *Prevotella* was significantly enriched during Vp infection in the mice in our study (Figure 5). This is consistent with previous studies that have suggested the *Enterobacteriaceae* family are more likely to increase as a result of colonization by enteric pathogens (Stecher et al., 2010; David et al., 2015). Besides, when the gut microbiota is perturbed so that it displays an abundance of the pathobiont *Prevotella*, this, along with induction of inflammation, is considered a cardinal marker of *Entamoeba histolytica* infection (Burgess and Petri, 2016). We hypothesize that *Prevotella* increased susceptibility to and colonization by Vp and therefore it has a strong correlation with Vp infection. In summary, both emergence or absence of intestinal bacterial communities can be associated with disease (Vigne et al., 2013; Xiong et al., 2015) and our microbiome profiling offered some feasible biomarkers for the diagnosis of Vp infection. If humans are infected with Vp, apart from using bacteriophage therapy to control and inhibit the virulence of *Vibrio* species (Letchumanan et al., 2016), adjusting the biomarker proportions of the species could also be used as a treatment.

There is evidence that changes in intestinal microbiota can significantly alter the biological functioning of the gut bacterial community in the host (Zhu et al., 2016). Consistent with this supposition, we found that many bacterial KEGG pathways were significantly different between the controls and Vp-infected mice (Figure 6). Infectious disease pathways, such as those used in *V. cholerae* and *H. pylori* infection, bacterial invasion of epithelial cells and epithelial cell signaling, were significantly enriched in Vp-infected mice compared to the controls. In contrast, the pathways involved in metabolism, digestion and cell growth decreased after Vp infection. Similarly, responses between infectious diseases and energy metabolism pathways were divergent in the gut microbiota of *Vibrio*-infected shrimp (Zhu et al., 2016). It has been proposed that changes in metabolic pathways are correlated with variations in their functions (Letchumanan et al., 2017). Therefore, Vp colonization markedly altered gut microbial function in the mice resulting in the effects on the progression of infection.

## Conclusion

In conclusion, this is the first attempt to explore the pathogenicity of Vp and its role in modulating gut microbiota. This study suggests that shifts in the make-up and quantity of gut microbiota populations play a potentially pathogenic role in Vp infection. These changes in the gut bacterial community lead to a change in gut microbe functions. This provides new insight for targeting interventions in response to changes in gut microbiota and improved understanding and prognosis of Vp infection. From a microbial ecological point of view, the findings reported here significantly improve our knowledge of how gut microbiota respond to Vp infection. However, the data in this study are derived from just a single sampling 12 h after infection, whereas mice intestinal communities are dynamic and change over time during the different stages of infection. Thus, a time-series design with multiple samplings is required to verify these ecological patterns in a real infection process.

## Data Availability Statement

The raw data supporting the conclusions of this article will be made available by the authors, without undue reservation, to any qualified researcher.

## Ethics Statement

The animal study was reviewed and approved by the College of Food Science and Technology, Guangdong Ocean University.

## Author Contributions

RW, LS, and YW designed the study. RW and YD performed the gut microbiota experiments and wrote the manuscript. QD and DS performed the pseudo germ-free mouse model. ZF and YW performed the statistical analyses. RG provided advice and revised the manuscript.

## Conflict of Interest

The authors declare that the research was conducted in the absence of any commercial or financial relationships that could be construed as a potential conflict of interest.

## Footnotes

1 http://huttenhower.sph.harvard.edu/galaxy/
